# The Pattern of Abdominal Ultrasound Scan Findings at the Department of Radiology at the University of Port Harcourt Teaching Hospital

**DOI:** 10.7759/cureus.76573

**Published:** 2024-12-29

**Authors:** Enighe Wananyo Ugboma, Ogheneochuko D Ray-Offor

**Affiliations:** 1 Radiology, University of Port Harcourt Teaching Hospital, Port Harcourt, NGA

**Keywords:** abdominal ultrasound, demographic patterns, diagnosis, findings, upth

## Abstract

Background: Abdominal ultrasound imaging is a standard diagnostic tool used in clinical practice. Understanding the patterns of sonographic findings in specific population demographics can lead to better clinical decisions and improved patient management. This study will evaluate the prevalent abdominal ultrasound scan findings and explore their demographic patterns based on age and sex characteristics at the University of Port Harcourt Teaching Hospital.

Objective: To evaluate the abdominal ultrasound findings and determine the relationship of these findings with the age and sex of patients.

Methods: A hospital-based retrospective study analyzed data from 681 patients who underwent abdominal ultrasound scans at the University of Port Harcourt Teaching Hospital using Epi-Info 7.1.4.

Results: The 681 participants' age distribution peaked in the 30-39 years category (24.7%). The sample included 48.6% males and 51.4% females. The most common ultrasound findings by category were hepatic disorders (n=173, 25.4%) and urogenital disorders (n=143, 21.0%). There were no significant differences in ultrasound findings across genders (p-values >0.05). Mean ages varied across categories, but no statistically significant age-related differences were detected (p-value>0.05).

Conclusion: The study gave insights into the demographic patterns of abdominal ultrasound findings at the University of Port Harcourt Teaching Hospital. Notably, there were high rates of hepatic and urogenital disorders. These findings will act as a guide for health professionals to understand common ultrasound findings in the abdomen.

## Introduction

Abdominal ultrasound imaging is an essential tool in modern diagnostic medicine, widely used for its safety, non-invasiveness, and cost-effectiveness. It uses high-frequency sound waves to produce images of organs and structures within the abdominal cavity [1]. It aids clinicians in diagnosing conditions such as gallstones, hepatic abnormalities, collecting system diseases, and vascular pathologies [1]. This modality is particularly beneficial in developing countries, including Nigeria, where resource constraints are a significant factor in diagnosis [2]. Ultrasound is important in clinical practice; it facilitates timely decision-making and intervention, thus improving patient outcomes. The reliance on ultrasound in thoracic and abdominal diagnosis has increased, contributing to better management strategies in healthcare settings [3]. Despite advances in imaging techniques such as computed axial tomography (CT) and magnetic resonance imaging (MRI), ultrasound remains the first-line investigation in many circumstances due to its accessibility, real-time imaging capabilities, and absence of ionizing radiation [4].

Various anatomical and pathological findings can be identified during an abdominal ultrasound. The most common indications for abdominal ultrasound include abdominal pain, suspected hernias, portal hypertension, evaluation of liver diseases, renal disorders, and other gastrointestinal abnormalities [5]. Understanding the prevalent findings can provide insights into a population's common health challenges and guide clinical and public health interventions. Previous studies have shown that demographic factors, including age and sex, can influence ultrasound findings [1]. For instance, certain conditions like gallbladder disease and liver pathologies can occur across different age groups and genders at varying frequencies. Recognizing these patterns is paramount for clinicians, as presentation variations affect diagnostic accuracy and management protocols [1].

This research is rooted in the need to fill the existing knowledge gap regarding the demographic patterns of abdominal ultrasound findings in the local context of the University of Port Harcourt Teaching Hospital (UPTH), which is a referral center with a large patient population, providing a representative sample for this study. Evaluating the abdominal ultrasound findings and the relationships with patients' age and sex will lead to obtaining data that could enhance diagnostic practices and patient management strategies with improved patient outcomes. Several studies have highlighted the impact of demographics on the prevalence and type of abdominal pathologies detected using ultrasound. Taher et al. reported a marked increase in hepatic disorders in older adults, aligning with the increasing incidence of liver diseases due to factors such as alcohol consumption, viral infections, and metabolic conditions [6]. Taher et al. noted that the prevalence of these conditions varies significantly across different age groups, necessitating demographic-specific diagnostic criteria and treatment approaches.

Furthermore, several studies show that many abdominal problems differ according to gender. For instance, gallstones are more common in women due to hormonal considerations, especially in the 20-40 age range [3]. Bailey et al., on the other hand, state that some conditions, such as ureteric calculi, are more common in men [6]. These results highlight the importance of stratifying demographic ultrasound findings to support precise diagnosis and efficient treatment. Gaining a comprehensive understanding of the impact gender plays in ultrasound findings can improve diagnostic accuracy and raise clinical awareness. In males, higher rates of urogenital pathologies, such as varicocele and testicular torsion, may affect referral practices and increase their likelihood of presenting for ultrasound evaluations [7]. In contrast, females are more likely to have ovarian cysts and pelvic inflammatory diseases, which present unique challenges and considerations in abdominal ultrasound documentation [8].

Furthermore, gender-based differences in hormonal levels, anatomical variations, and lifestyle factors can lead to disparities in the prevalence of specific pathologies. For instance, diseases influenced by hormonal levels, such as polycystic ovarian syndrome, are more common in females [7]. In contrast, lifestyle-related conditions like non-alcoholic fatty liver disease tend to be more prevalent in males [8]. These disparate findings underscore the need for tailored screening practices and diagnostic considerations in clinical settings. Age is a factor that significantly influences the presentation and severity of abdominal diseases, as age-related changes in anatomy and physiology can lead to varying ultrasound presentations [8]. For example, as individuals age, the incidence of liver diseases, including liver cirrhosis and hepatocellular carcinoma, increases substantially due to cumulative risk factors. Research has shown that early detection through appropriate imaging can significantly improve prognosis, highlighting the importance of age in disease management [8].

Additionally, developmental changes in younger populations, such as in children, can lead to unique ultrasound findings. Conditions like intussusception and appendicitis often present differently in pediatric cases compared to adults, necessitating specific considerations in ultrasound imaging for different age groups [3]. Healthcare providers must recognize these age-related trends to optimize diagnostic accuracy and patient care.

In Nigeria, limited access to advanced imaging modalities like CT and MRI has resulted in a heavy reliance on ultrasound for abdominal assessments [2]. Understanding the local prevalence of abdominal pathologies through ultrasound is critical for healthcare resource allocation, public health planning, and further research initiatives. As healthcare systems strive for improvement amid existing challenges, data from studies like this can provide valuable insights into the most common conditions affecting the populace. Several studies have aimed to map the landscape of ultrasound findings in the Port Harcourt region. Nevertheless, a notable gap in comprehensive data encompasses the prevalence of abdominal pathologies and their demographic associations [2]. This study seeks to address this gap by providing a detailed account of the patterns of abdominal ultrasound scan findings about age and sex in a local population. The findings of this study will have implications not only for clinical practice but for the academic community. By documenting the prevalent abdominal ultrasound findings and their correlations with demographic factors, this research can contribute to the body of knowledge necessary for improving diagnostic accuracy in ultrasound examinations. The insights gained could inform the training of radiologists and sonographers, enhancing their awareness of demographic influences on abdominal conditions. Furthermore, this study will serve as a foundation for future research directions. Establishing a database for abdominal ultrasound findings can facilitate further studies aimed at assessing trends over time, investigating the effectiveness of ultrasound in identifying specific pathologies, or comparing regional variations in ultrasound findings [5].

In summary, the introduction of this study highlights the significance of understanding the patterns of abdominal ultrasound findings within the context of demographic factors such as age and sex. Given ultrasound's vital role in the diagnostic landscape, particularly in regions with limited access to advanced imaging, this research aims to provide valuable insights that can inform clinical practice, enhance diagnostic accuracy, and guide public health interventions. By exploring the relationship between ultrasound findings and demographic characteristics, we hope to contribute essential data to improve healthcare outcomes in the University of Port Harcourt Teaching Hospital and similar settings.

## Materials and methods

Study design and methods

This study employed a hospital-based retrospective design. It analyzed records from the Radiology Department of UPTH to evaluate the patterns of abdominal ultrasound scan findings.

Using Cochrane's formula, a minimum sample size of 665 was determined based on a 95% confidence level, a power of 90%, and a proportion of previous abdominal ultrasound requests at 19.7% (0.197), as reported by Onwuchekwa & West [9]. Data from 681 participants who underwent abdominal ultrasound scans between January 2022 and December 2023 were included to ensure representativeness.

The inclusion criteria for this study comprised all patients who underwent abdominal ultrasound scans at the Radiology Department of the UPTH between January 2022 and December 2023. Participants of all ages were eligible, provided their medical records contained complete demographic data, including age, sex, and documented ultrasound findings. Cases performed that adhered to the hospital's diagnostic protocols, and consent for using anonymized patient data for the research was required for inclusion, as approved by the UPTH Ethical Review Committee (Protocol number: UPTH/ADM/90/5.11/VOL.XI/1760).

Exclusion criteria included patients with incomplete medical records, such as missing demographic details or ultrasound findings. Patients who underwent non-abdominal ultrasounds, such as cardiac or obstetric scans, were excluded, as were cases where multiple abdominal scans were performed on the same patient. The first scans were included to avoid data duplication. Ultrasounds with poor imaging quality or inadequate documentation and patients or guardians who did not consent to anonymized data for research were excluded.

Data collection

Demographic data, including patient age and sex, along with ultrasound findings, were extracted from the medical records of the Radiology Department. Each ultrasound scan was conducted by licensed radiologists following the hospital's established protocols. The records ensured that all relevant clinical details were accurately documented, maintaining the integrity of the data used in the study. Ultrasound findings were grouped into six main groups: normal findings, hepatic disorders, urogenital disorders, respiratory disorders, cardiovascular disorders, and gastrointestinal disorders. This classification allowed for a structured analysis of the types and frequency of abnormalities identified in the abdominal ultrasound scans, ensuring comprehensive coverage of the relevant medical conditions within the study population.

Data analysis

Data analysis was conducted using Epi-Info 7.1.4 (CDC, USA). Categorical variables were expressed as absolute and relative frequencies, while numerical variables were summarized using means and standard deviations. Chi-square tests were employed to determine significant relationships in ultrasound findings by sex. Analysis of Variance (ANOVA) was utilized to compare mean ages across different categories of ultrasound findings. Statistical significance was defined as P<0.05.

Ethical considerations: Ethics approval was obtained from the UPTH Ethical Review Committee, and patient confidentiality was maintained throughout the study.

## Results

Age and sex distribution

The study included a total of 861 patients with refractive anomalies. The age distribution shows the highest proportion of participants in the 30-39 years category (24.7%, 168 individuals), followed by those aged 40-49 years (15.7%, 107 individuals) (Table 1). The younger age groups (under ten years, 13.7%) and older age groups (≥90 years, 0.1%) are represented minimally. Regarding sex distribution, there were 350 females (51.4%) and 331 males (48.6%), indicating a slight female predominance. The data suggest that refractive anomalies are more prevalent among middle-aged individuals than younger or older populations.

**Table 1:** Age and sex distribution of the respondents

Variables	Frequency	Percentage
Age category		
<10 years	93	13.7
10 – 19 years	66	9.7
20 – 29 years	61	9.0
30 – 39 years	168	24.7
40 – 49 years	107	15.7
50 – 59 years	84	12.3
60 – 69 years	67	9.8
70 – 79 years	25	3.7
80 – 89 years	9	1.3
≥90 years	1	0.1
Sex		
Male	331	48.6
Female	350	51.4

Distribution of ultrasound scan findings

**Figure 1:**
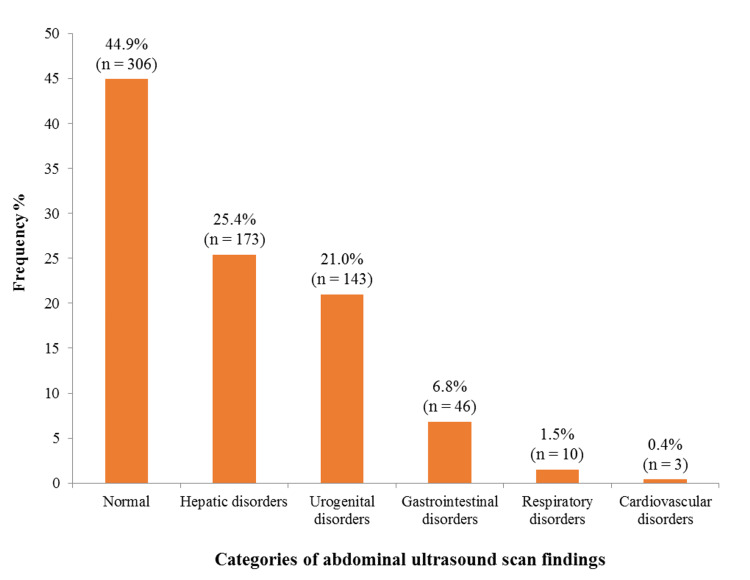
Distribution of categories of ultrasound scan findings among study participants

Figure 1 illustrates the distribution of ultrasound findings among participants with refractive anomalies. Although specific categories are not detailed here, the figure indicates the relative prevalence of various conditions identified through ultrasound. Typical categories may include normal findings, hepatic disorders, urogenital disorders, and gastrointestinal disorders. This distribution emphasizes the diagnostic utility of abdominal ultrasound in identifying prevalent health conditions among the study participants. The findings suggest the importance of ultrasound in assessing refractive anomalies and associated abdominal pathologies impacting this demographic.

Comparison of abdominal ultrasound scan findings by gender of study participant

Table 2 compares the ultrasound findings based on gender. The results indicate that males had a slight majority in the normal category (51.3%) and hepatic disorders (53.8%), whereas females had a higher prevalence of urogenital disorders (53.1%). For respiratory disorders, both genders had equal representation (50%). Cardiovascular disorders were exclusively found in males (100%), while gastrointestinal disorders showed a similar distribution (54.3% males vs. 45.7% females). The p-values for all categories indicate no statistically significant differences, suggesting that the prevalence of ultrasound findings is comparable among genders within this study population.

**Table 2:** Comparison of abdominal ultrasound scan findings by gender

	Gender				
Categories of abdominal ultrasound scan findings	Male n (%)	Female n (%)	M:F	Chi-square for homogeneity	p-value
Normal	157 (51.3)	149 (48.7)	1.1:1	0.209	0.647
Hepatic disorders	93 (53.8)	80 (46.2)	1.2:1	0.977	0.323
Urogenital disorders	67 (46.9)	76 (53.1)	0.9:1	0.566	0.452
Respiratory disorders	5 (50.0)	5 (50.0)	1:1	0.000	1.000
Cardiovascular disorders	3 (100.0)	0 (0.0)	3:0	3.000	0.083
Gastrointestinal disorders	25 (54.3)	21 (45.7)	1.2:1	0.348	0.555

Comparison of mean ages across abdominal ultrasound scan findings

**Figure 2:**
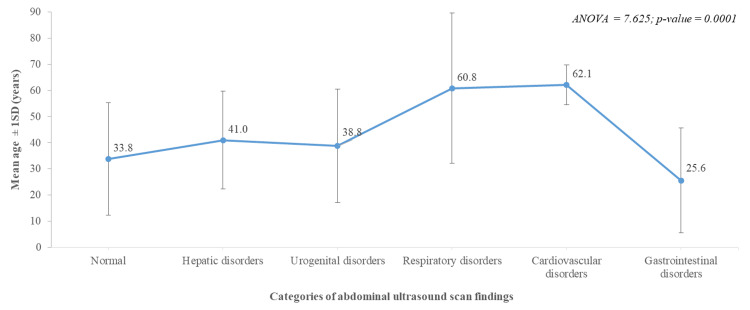
Error bar showing mean ages of study participants across the categories of abdominal ultrasound scan findings

Figure 2 presents the mean ages of participants across different categories of abdominal ultrasound findings, indicated by error bars reflecting variability. If error bars overlap significantly, it suggests that age differences for specific conditions may be minimal or insignificant. Conversely, distinct mean age distributions for specific pathologies could indicate age-related health trends. This analysis is crucial in understanding which age groups may be more susceptible to specific abdominal conditions.

## Discussion

Abdominal ultrasound has emerged as an essential diagnostic tool due to its non-invasive nature, effectiveness, and safety profile, particularly in under-resourced regions. The current study at the University of Port Harcourt Teaching Hospital (UPTH) aimed to elucidate the patterns of abdominal ultrasound findings while examining the influence of demographic factors such as age and sex. By analyzing 681 ultrasound examinations, the findings provide vital information about prevalent abdominal conditions in this context, contributing to clinical practices and broader public health initiatives in Nigeria. The analysis revealed that most participants were aged between 30-39 years (24.7%) and 40-49 years (15.7%), indicating the relevance of focusing healthcare resources on these demographics. The sample showed a slight female predominance (51.4%), with significant rates of hepatic disorders (25.4%) and urogenital disorders (21.0%). Despite the subtle variations in ultrasound findings based on gender, the differences were not statistically significant, highlighting a uniform distribution of abdominal conditions across both sexes.

The findings align with a broader trend observed in various studies across Africa. A study in Cameroon [10] found that hepatic disorders are common in middle-aged populations, primarily linked to viral hepatitis and alcohol consumption, mirroring the findings from Port Harcourt. The high incidence of liver pathology in regions with similar socio-economic factors emphasizes the importance of understanding these health challenges within a developing nation context. Furthermore, the burden of liver diseases in Sub-Saharan Africa has been associated with the rise of metabolic syndromes exacerbated by dietary habits and increasing lifestyle-related diseases [11]. Research conducted by Moussavou-Boundzanga et al. in Gabon reinforces the need to screen for hepatic disorders in young adults, as liver disease has emerged as a leading cause of morbidity [12]. These findings resonate with the context in Port Harcourt, reinforcing the need for targeted screening initiatives to combat rising liver disease rates.

Gender disparities in abdominal ultrasound findings have also been well-documented globally, contributing to our understanding of how such differences manifest in different populations. For instance, a study in Kenya reported that women were more likely to present with gynecological-related abdominal conditions, reflecting hormonal influences and lifestyle factors that differ by gender [13]. This finding aligns with the Port Harcourt study, where urogenital disorders were more prevalent in females (53.1%), suggesting a need for gender-specific health education and awareness campaigns. Conversely, studies in Tunisia indicated that males exhibited a higher prevalence of gallstones and ureteric calculi, suggesting that lifestyle behaviors, such as diet and hydration, significantly influence abdominal pathologies [14]. The consistency of such findings across various African contexts highlights the necessity for tailored diagnostic approaches and public health strategies that recognize these demographic differences.

The findings from this study are crucial for several reasons. Firstly, they underscore the role of abdominal ultrasound in diagnosing common conditions within a specific population where advanced imaging techniques are often inaccessible. Given that Nigeria has limited healthcare resources, increasing reliance on ultrasound enhances timely interventions that could mitigate severe health outcomes. Furthermore, UPTH serves a diverse patient population as a major referral center. The study's findings on common abdominal conditions provide valuable reference points for radiologists and healthcare practitioners in the region, enabling them to tailor diagnostic approaches based on prevalent pathologies. Knowledge of common ultrasound findings can significantly improve diagnostic accuracy and prompt appropriate management based on demographic characteristics.

Additionally, this study draws attention to public health issues specific to Nigeria. Given the increased incidence of hepatic and urogenital disorders found in this study, it underscores the urgent need for public health campaigns aimed at prevention, awareness, and early detection. The results could inform policymakers and health organizations about critical areas requiring intervention, thereby effectively guiding resource allocation. Despite its contributions, the study has limitations. The retrospective nature of data collection inherently introduces biases related to incomplete records or inconsistencies in documentation. This may affect the reliability of the findings, particularly in interpreting the prevalence of specific conditions. Furthermore, as the study was confined to a single center, it may not represent the entire region or country, leading to limitations in generalizability.

The research also focuses solely on ultrasound findings while neglecting associated demographic factors such as socio-economic status, dietary patterns, and comorbidities, significantly affecting diagnosis and treatment outcomes [15]. A critical gap in knowledge exists regarding lifestyle factors, education levels, and healthcare-seeking behaviors, which could influence the prevalence and presentation of abdominal conditions in diverse populations. Consequently, future studies would benefit from exploring these variables more holistically. The study addresses gaps by providing a structured analysis of demographic factors influencing ultrasound findings in a specific Nigerian context. The documentation of prevalent abdominal conditions serves not only to enhance local knowledge but also to contribute to the global understanding of health issues affecting similar populations.

This research highlights the trends of hepatic and urogenital disorders, providing foundational data that can inform future studies and interventions. Establishing a local database on abdominal ultrasound findings represents a significant advancement in public health monitoring, facilitating a deeper understanding of health trends over time. Future research should adopt a prospective multi-center design encompassing a broader demographic and exploring the lifestyle factors and healthcare-seeking behaviors influencing ultrasound findings, as these elements are vital in shaping health outcomes. Longitudinal studies could track changing trends in abdominal conditions, equipping healthcare providers with real-time insights to adapt management strategies. Comparative studies across different regions could also enhance understanding of the demographic trends impacting ultrasound findings, particularly in African countries facing similar health challenges.

Integrating advanced imaging techniques alongside traditional methods can further enhance diagnostic effectiveness. While ultrasound remains the primary imaging modality in resource-limited settings, incorporating other technologies can potentially improve diagnostic accuracy and patient care outcomes. The study at the University of Port Harcourt Teaching Hospital offers valuable insights into the region's demographic factors influencing abdominal pathologies. By emphasizing the significant trends in hepatic and urogenital disorders, it underscores the urgent need for targeted public health interventions and educational initiatives. Moreover, this research helps establish a localized database for abdominal ultrasound findings, paving the way for future studies and strengthening the diagnostic capabilities of healthcare providers. A holistic approach incorporating demographic factors, lifestyle influences, and advanced imaging techniques will be crucial in effectively addressing the healthcare challenges faced in Nigeria and beyond.

Limitations

The study's retrospective design may introduce biases due to incomplete records or inconsistencies in documentation, and findings from a single-center study may limit generalizability to broader Nigerian populations. The research focuses exclusively on ultrasound findings, neglecting other relevant demographic factors such as socio-economic status, dietary habits, comorbidities, key lifestyle factors, and healthcare-seeking behaviors influencing abdominal conditions that were not explored in depth, which could have provided a more comprehensive understanding of the findings. 

Recommendations

Implement targeted public health campaigns focused on liver health and urogenital awareness, particularly within the middle-aged demographic. Develop a database of ultrasound findings to comprehensively understand abdominal pathologies across Nigeria. Encourage the integration of lifestyle factors and socio-economic status into future studies to better understand their impact on health outcomes, promote the training of healthcare practitioners on gender-specific health considerations in abdominal ultrasound diagnostics, and foster collaborations between healthcare facilities to share data and best practices for improved patient management strategies.

## Conclusions

This study at the UPTH analyzed 681 abdominal ultrasound examinations, revealing a high prevalence of hepatic and urogenital disorders, with most patients being in the middle-aged group. There was a slight predominance of female patients, though the difference between genders was not statistically significant. The findings align with similar studies in Sub-Saharan Africa, highlighting the role of lifestyle factors in liver disease. The study underscores the value of ultrasound in diagnosing conditions in resource-limited settings and advocates for public health initiatives focused on liver and urogenital health, particularly for middle-aged populations in Nigeria.
